# Evaluation of AGP Fucosylation as a Marker for Hepatocellular Carcinoma of Three Different Etiologies

**DOI:** 10.1038/s41598-019-48043-1

**Published:** 2019-08-09

**Authors:** Jing Liang, Jianhui Zhu, Mengmeng Wang, Amit G. Singal, Mobolaji Odewole, Sofia Kagan, Veronica Renteria, Suyu Liu, Neehar D. Parikh, David M. Lubman

**Affiliations:** 1 Department of Surgery, University of Michigan, Ann Arbor, MI 48109 USA; 2 School of Chemical Engineering and Technology, China University of Mining and Technology, Xuzhou, Jiangsu 221116 China; 3 Department of Emergency Medicine, Jinling Hospital, Medical School of Nanjing University, Nanjing, Jiangsu 210002 China; 4 Department of Internal Medicine, University of Texas Southwestern Medical Center, Dallas, TX 75390 USA; 5 Department of Biostatistics, University of Texas MD Anderson Cancer Center, Houston, TX 77030 USA; 6 Department of Internal Medicine, University of Michigan, Ann Arbor, MI 48109 USA

**Keywords:** Hepatocellular carcinoma, Diagnostic markers

## Abstract

A mass spectrometric analysis platform has been developed to determine whether glycosylation patterns of alpha-1 acid glycoprotein (AGP) could be used as a marker for early detection of hepatocellular carcinoma (HCC) in different etiologies, i.e. non-alcoholic steatohepatitis (NASH), alcoholic liver disease (ALC), and hepatitis C virus (HCV). MALDI-MS profiling of *N*-glycans of AGP purified from 20 μL of patient serum in HCC (n = 72) and liver cirrhosis (n = 58) showed that a unique trifucosylated tetra-antennary glycan (*m/z* 3490.76) was predominantly identified in HCCs but was absent in healthy subjects and the majority of cirrhosis patients. Receiver operation characteristic (ROC) curve analysis showed that the trifucosylated *N*-glycan of AGP (triFc_AGP) could differentiate HCC from cirrhosis with an area under the curve (AUC) of 0.707, 0.726 and 0.751 for NASH, ALC and HCV, respectively. When combining triFc_AGP with INR and AFP, the panel had the greatest benefit in detection of NASH-related HCCs, with a significantly improved AUC of 0.882 for all NASH HCCs and 0.818 for early NASH HCCs compared to AFP alone (0.761 and 0.641, respectively). Moreover, triFc_AGP could serve as a potential marker for monitoring AFP-negative HCC patients.

## Introduction

Liver cancer has the second highest worldwide cancer mortality rate with 700,000 annual deaths recorded globally in recent years^1^. Hepatocellular carcinoma (HCC), the predominant form of liver cancer, occurs mainly in patients with cirrhosis from various etiologies including viral hepatitis, alcoholic liver disease (ALC)^2^, and non-alcoholic steatohepatitis (NASH)^3^. Approximately 80–90% of HCC patients have underlying liver cirrhosis^4^. While early stage HCC has many curative treatment options, late stage HCC has a high associated mortality, due to limited efficacy of HCC systemic therapies^5,6^.

Guidelines from the American Association for the Study of Liver Disease (AASLD) recommend HCC surveillance with abdominal ultrasound every 6 months with or without alpha fetoprotein (AFP) in patients with cirrhosis^7^. However, current HCC surveillance strategies have limited sensitivity in detecting early stage HCC and can also suffer from poor specificity^8,9^. Thus, accurate and sensitive diagnostic techniques for screening in patients with liver cirrhosis to detect early-stage HCC are urgently needed. Serum biomarkers have been developed to aid in the early detection of HCC^10–13^. AFP is the most common serum biomarker used for diagnosis and surveillance of HCC, however, the sensitivity and the predictive value of the AFP-based test are still insufficient for early HCC detection^8^.

Alpha-1-acid glycoprotein (AGP), a serum glycoprotein with 45% carbohydrate content attached in the form of five *N*-linked complex glycans^14^, is an acute phase protein synthesized mainly by hepatocytes. Acute phase proteins (APPs) are a group of proteins whose serum concentration changes in response to an acute phase, i.e. intense inflammation or tissue destruction. During an acute phase response, changes include not only the protein level of AGP but also its glycosylation patterns^15^, whereas the protein synthesis and glycosylation of AGP are independently regulated^16,17^. Chronic liver disease and cirrhosis have been shown to affect the expression of AGP^18^ and its glycosylation changes in both branching degree and fucosylation degree. Aberrant glycosylation of AGP is highly related to the carcinoma progression of HCC^19–21^, especially with elevated fucosylation^22^. However, it is still not clear how AGP glycoforms alter during the progression from liver cirrhosis to HCC of different etiologies.

Cancer-associated glycosylation aberrations provide a rationale for discovering new biomarkers using glycomic/glycoproteomic technologies^23–28^. Previous studies revealed that although there was an increase in fucosylation level in both cirrhosis and HCC patients, HCC patients expressed AGP with more multifucosylated glycans^19,20^. Zhang *et al*. reported increased levels of fucosylation in AGP in patients with liver cirrhosis and HCC compared to healthy controls, where different degrees of fucosylation could distinguish HCC from cirrhosis^21^. These studies indicated that the fucosylation pattern of AGP is unique in patients with HCC, thus determination of the specific changes in glycan structures in AGP could become a sensitive biomarker for detecting early HCC.

Recently developed mass spectrometric techniques in glycoproteomics have contributed a great deal in comprehensive analyses of glycans expressed in glycoproteins. Among them, matrix-assisted laser desorption ionization-mass spectrometry (MALDI-MS) has the advantages of high throughput, high sensitivity, rapid operation, and minimal fragmentation of the targeted molecules, which can be employed to provide structural information of glycoproteins^29,30^. Although glycans released from their host proteins lose site-specific information, they provide a much simpler pattern of mass spectra than glycopeptides. A comparison of different glycoforms of AGP characterized by MALDI-MS would provide information about its potential value as a biomarker.

The aim of this analysis is to develop a mass spectrometric platform to determine the alterations in *N*-glycan structures of serum AGP between HCC and liver cirrhosis patients in various etiologies, and to further evaluate whether the altered glycosylation patterns of AGP could serve as a marker for early detection of HCC. For this purpose, we purified AGP from serum in patients with HCC and liver cirrhosis of the three common etiologies in the United States, HCV, ALC and NASH, respectively, followed by release of *N*-glycans and MALDI-MS profiling. Using a MALDI-MS based quantitative method^31^, we compared *N*-glycan structures of serum AGP among individual patients to investigate the alterations in *N*-glycan patterns correlated with patient disease status and specific etiologies.

## Results and Discussion

**Figure 1:**
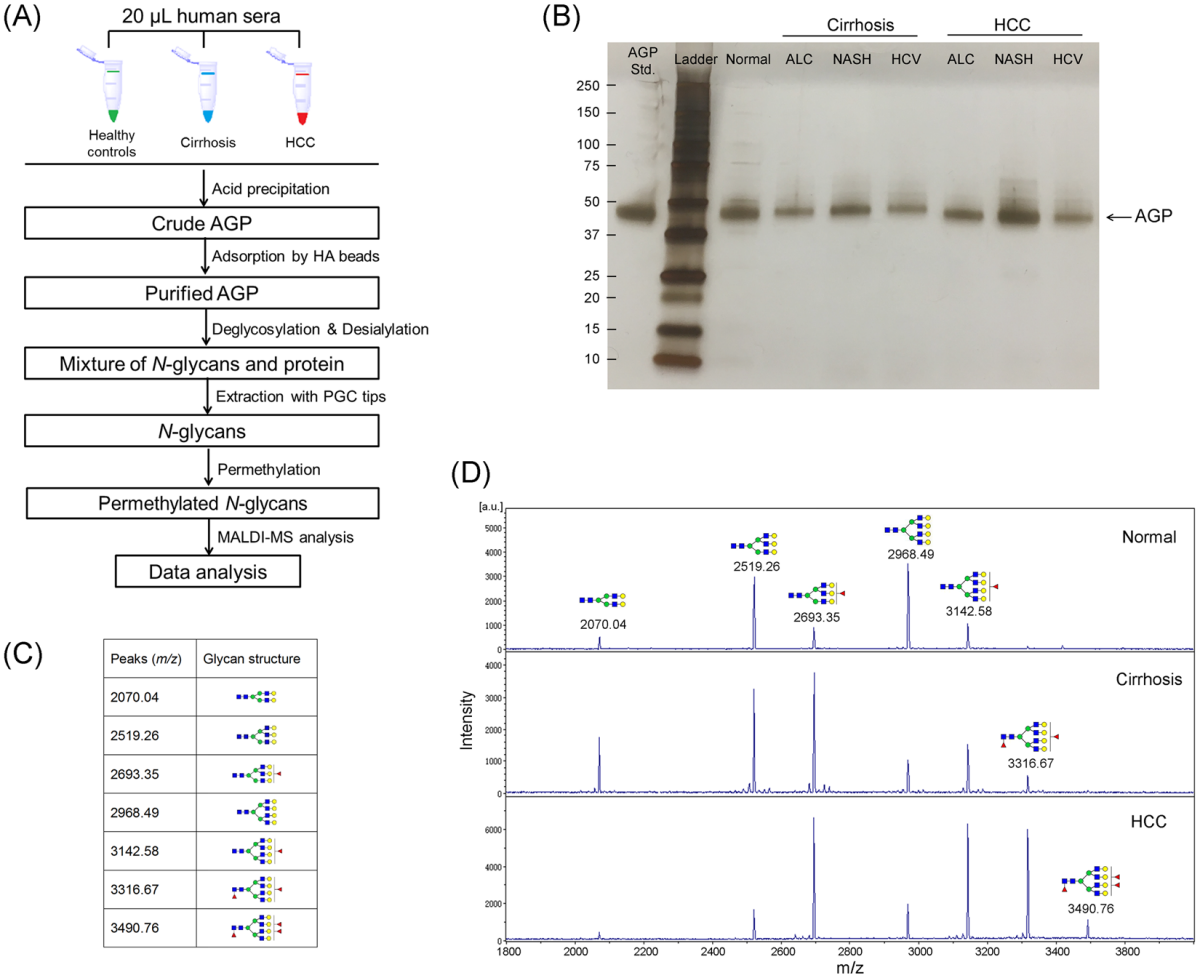
(**A)** Workflow of *N*-glycan profiling of alpha-1-acid glycoprotein (AGP) derived from sera of healthy controls, liver cirrhosis and HCC. (**B)** SDS-PAGE image of purified AGP from patient serum in HCC and cirrhosis of different etiologies, i.e. ALC, NASH, and HCV, respectively, with 0.4 μg of an AGP standard protein as a reference. **(C)** 7 main Desialylated *N*-Glycans of serum AGP identified in this study. **(D)** Representative MALDI-TOF MS spectra of desialylated *N*-glycans in serum AGP derived from healthy controls and patients with liver cirrhosis and HCC, respectively. The bifucosylated tetra-antennary glycan (*m/z* 3316.67) was highly elevated in HCC and cirrhosis compared to normal subjects. The trifucosylated tetra-antennary glycan (*m/z* 3490.76) was predominantly identified in HCC patients but was absent in normal subjects and the majority of cirrhosis samples. (The symbols used in the structural formulas as: red triangle, Fuc; blue square, GlcNAc; green circle, Man; yellow circle, Gal).

The workflow of this study is summarized in Fig. 1A. In total, 36 NASH-, 39 ALC-, and 55 HCV-related patients with cirrhosis and 7 healthy controls were included in this study. The clinical information is summarized in Table 1. There were 72 HCC cases (17 NASH-, 20 ALC-, and 35 HCV-related, respectively), and 58 cirrhosis (19 NASH-, 19 ALC-, and 20 HCV-related, respectively). Age was moderately higher in HCC (62.9 y) compared to cirrhosis patients (58.0 y). As expected, values of AFP were significantly higher for HCC patients than corresponding cirrhosis patients for each etiology, with median values of 7.2 vs. 3.4 (ng/mL) in NASH (P = 0.0086), 5.9 vs. 2.6 in ALC (P = 0.0006), and 33.0 vs. 4.1 in HCV (P < 0.0001), respectively. AFP values in HCV-related HCC patients was significantly higher than that of ALC- and NASH-related HCC patients (P = 0.0149). There was also a significant difference in INR between HCC and cirrhosis (P = 0.0266).

**Table 1:** Summary of Sample Clinical Characteristics.

Diagnostic States	HCC			Cirrhosis		
Number	72			58		
Age (year, mean ± SD)	62.9 ± 9.4			58.0 ± 7.7		
Gender (F/M)	23/49			27/31		
**Etiology**	**NASH** ^**a**^ **(n = 17)**	**ALC** ^**a,b**^ **(n = 20)**	**HCV** ^**b**^ **(n = 35)**	**NASH** ^**a**^ **(n = 19)**	**ALC** ^**a,b**^ **(n = 19)**	**HCV** ^**b**^ **(n = 20)**
ALT^c^	38.4 ± 22.6	38.8 ± 28.6	89.1 ± 49.5	33.3 ± 14.7	36.6 ± 17.6	79.9 ± 48.4
AST^c^	62.3 ± 35.6	60.8 ± 36.3	108.2 ± 50.6	45.7 ± 15.8	55.9 ± 44.2	104.3 ± 73.3
Albumin (g/dL)^c^	3.6 ± 0.6	3.3 ± 0.7	3.6 ± 0.6	3.7 ± 0.6	3.6 ± 0.6	3.3 ± 0.7
TBili (mg/dL)^c^	1.2 ± 1.0	2.4 ± 3.9	1.8 ± 3.3	1.2 ± 1.0	1.5 ± 1.1	1.6 ± 0.9
INR^c^	1.0 ± 0.13	1.2 ± 0.18	1.1 ± 0.21	1.2 ± 0.20	1.1 ± 0.15	1.2 ± 0.14
MELD^c^	8.4 ± 2.9	11.2 ± 4.6	9.3 ± 3.7	10.9 ± 5.0	9.0 ± 3.4	10.6 ± 2.6
AFP^c^ (ng/mL, median)	7.2	5.9	33.0^d^	3.4^e^	2.6	4.1
AFP < 20 ng/mL (%)	70.6%	80%	44.1%	94.4%	100%	90.0%
TNM stage (I/II/III/IV)	8/3/3/3	6/5/6/3	6/12/7/10	NA	NA	NA
Early HCC (%)	64.7%	55.0%	51.4%	NA	NA	NA

^a^Samples were provided by the UT Southwestern Medical Center, Dallas, TX.

^b^Samples were provided by the Division of Gastroenterology, University of Michigan, Ann Arbor, MI. NASH, nonalcoholic steatohepatitis; ALC, alcohol consumption; HCV, hepatitis C virus.

^c^ALT, AST, Albumin, TBili, INR, MELD, and AFP values were provided by the Hospitals. ALT, alanine aminotransferase; AST, aspartate aminotransferase; TBili, total bilirubin; INR, international normalized ratio; MELD, model for end-stage liver disease. Values are presented as mean ± SD or median.

^d^One patient with HCC induced by HCV had AFP missing.

^e^One patient with Cirrhosis induced by NASH had AFP missing.

### ELISA determination of AGP in serum

First, we evaluated the protein level of AGP in individual patient sera using a human AGP ELISA Kit (abcam, Cambrige, MA). The scatter plot of serum AGP concentration in patients with HCC or cirrhosis of different etiologies is shown in Supplemental Fig. S1.

The serum AGP concentration (mean ± SD) in the NASH, ALC, and HCV patients was 1339 ± 618, 1108 ± 528, and 858 ± 433 μg/mL, respectively. ANOVA analysis among the three disease groups showed the NASH subgroup had a significantly higher level of serum AGP than the HCV subgroup (P = 0.0028) in this sample set.

As shown in Fig. S1, the serum AGP concentrations in NASH-HCC vs. NASH-Cirrhosis, ALC-HCC vs. ALC-Cirrhosis, HCV-HCC vs. HCV-Cirrhosis patients were 1578 ± 704 vs. 1124 ± 446 (P = 0.0255), 1182 ± 618 vs. 1033 ± 439 (P = 0.5418), and 827 ± 336 vs. 919 ± 600 μg/mL (P = 0.5903), respectively. When compared across the etiologies, NASH-HCC patients had a significantly higher level of serum AGP than HCV-HCC (P = 0.0008) and HCV-Cirrhosis (P = 0.0351) patients.

As an acute phase protein, AGP serum concentration changes have been correlated with increases in hepatic synthesis. Expression of the AGP gene is regulated by glucocorticoids and a cytokine network consisting of interleukin-1β (IL-1β), tumor necrosis factor-α (TNFα), interleukin-6 and IL-6 related cytokines^14^. However, the biological mechanism for this regulation is not well understood.

In the following quantitative mass spectrometry analyses, the abundance of each *N*-glycan of AGP was normalized by the sum of the abundances of all *N*-glycans identified in each sample. The normalization procedure can reduce the effect of the variation on AGP protein abundance among samples. Thus, the changes in the fucosylation level of serum AGP determined in this study were due to the specific glycosylation alteration rather than protein abundance variation.

### Purification of serum AGP

AGP is a relatively abundant protein with a very low pI of 2.8~3.8, which makes it possible to isolate AGP from serum by a pH based precipitation method^32^. This step was performed within 20 s, which can precipitate most of the impurity proteins while AGP remains in the liquid phase. Further treatment with anti-HA beads yield a high purity of AGP which was confirmed by gel electrophoresis with silver staining. As shown by SDS-PAGE (Fig. 1B), one-tenth of the AGP, isolated from the sera of cirrhosis and HCC patients induced by NASH, ALC, and HCV, respectively, was loaded on the gel, where 0.4 μg of standard AGP (Abcam, Cambridge, MA) was employed as a positive control. Only one band with a molecular weight of 45 kDa was present, corresponding to AGP protein.

With two steps of purification, AGP was isolated from serum, whose purity can meet the requirements of *N*-glycan analysis. Thus, this optimized method made it possible to isolate AGP from a large number of serum samples.

### *N*-Glycan profiles of AGP with desialylation

Figure 1C summarizes the 7 desialylated *N*-glycans of serum AGP identified in this study. Representative MALDI-MS spectra of the desialylated *N*-glycans of serum AGP in healthy controls and patients with liver cirrhosis or HCC, respectively, are shown in Fig. 1D. *N*-glycomic analysis revealed 7 main *N*-glycan structures of the desialylated AGP in MALDI-MS spectra, including non-fucosylated bi-, tri- and terta-antennary *N*-glycans (*m*/*z* 2070.07, 2519.28, and 2968.49, respectively), mono-fucosylated tri- and tetra-antennary glycans (*m*/*z* 2693.40 and 3142.69), and bi- and tri-fucosylated tetra-antennary glycans (*m*/*z* 3316.69 and 3490.76). For some samples, mono-fucosylated biantennary glycan (*m*/*z* 2244.13) and bi-fucosylated triantennary glycan (*m*/*z* 2867.48) in low abundance were observed in the spectra, while for most of the samples these two peaks were absent. The structural information on branching degree and core or antennary fucosylation were further determined by MALDI-QIT-TOF MS/MS analysis, as shown in the Supplemental Fig. S2.

When comparing the spectra of healthy controls, cirrhosis and HCC patients (Fig. 1D), we observed that for normal samples, the nonfucosylated tri- and tetra-antennary peaks were most abundant, while the nonfucosylated biantennary and fucosylated tri- and tetra-antennary peaks were expressed in much lower abundance. However, in cirrhosis and HCC samples, the intensity of nonfucosylated bi-, tri-, and tetra-antennary *N*-glycans decreased at different levels compared to normal samples. Notably, the fucosylated glycans showed a significant increase in cirrhosis and HCC samples, especially the bifucosylated tetra-antennary *N*-glycan (*m*/*z* 3316.69), which was absent in most of the normal samples but present in low abundance in cirrhosis cases and was markedly increased in HCC cases. This structure can be used to distinguish cirrhosis and HCC patients from healthy controls. A unique pattern of fucosylated *N*-glycans present on AGP was the trifucosylated tetra-antennary *N*-glycan (*m*/*z* 3490.76), which was absent in most of the cirrhosis samples but expressed in the majority of HCC cases. This tri-fucosylated glycan structure may serve as a unique potential marker for HCC cases. Moreover, the significantly decreased nonfucosylated *N*-glycans and significantly increased fucosylated tetra-antennary structures were characteristic of the mass spectra of the HCC samples compared to that of cirrhosis.

### Fucosylation correlation with etiology

**Figure 2:**
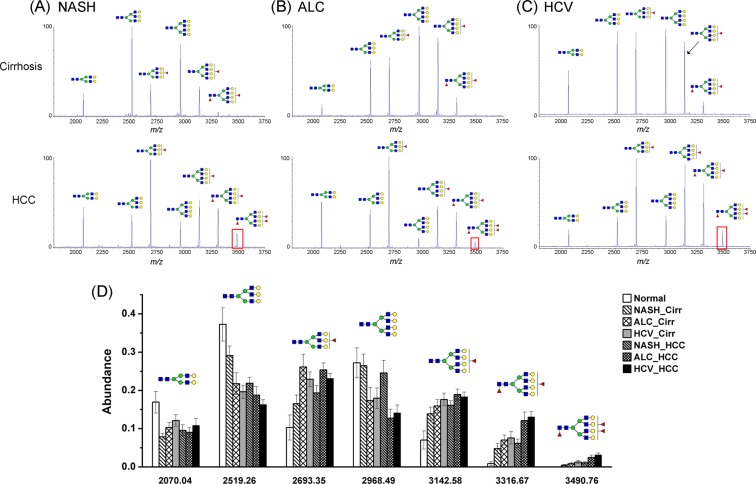
(**A**–**C**) MALDI- TOF MS spectra showing the difference of glycosylation in *N*-glycans of AGP between HCC and cirrhosis in relation to the etiology, NASH (**A**), ALC (**B**), and HCV (**C**), respectively. The elevated presence of the trifucosylated tetra-antennary glycan in HCCs compared to cirrhosis of each etiology is highlighted with a red rectangle. **(D)** Relative abundance alteration of 7 main *N*-glycans in serum AGP derived from healthy controls and patients with cirrhosis and HCC of different etiologies, respectively.

Since HCC samples showed a distinct elevation in both bi- and tri-fucosylated tetra-antennary glycans compared to cirrhosis samples, we further investigated the alteration in fucosylation between HCC and cirrhosis patients in each etiology. In Fig. 2 is shown the zoomed-in MS spectra of *N*-glycans on serum AGP in cirrhosis and HCC cases induced by NASH (A), ALC (B), and HCV (C), respectively.

For NASH samples (Fig. 2A), we observed a decrease in non-fucosylated tri- and tetra-antennary *N*-glycans, and an increase in all the fucosylated *N*-glycans especially in the bi- and tri-fucosylated tetra-antennary structures in HCC samples compared to cirrhosis. The trifucosylated tetra-antennary peak (*m*/*z* 3490.76), which was absent in 10 of 19 NASH-related cirrhosis samples but present in all the NASH-related HCC samples (highlighted with a red rectangle), resulted in an expression in NASH cirrhosis samples with a mean value of 0.5% as compared to 1.1% in NASH HCC samples (Table S1). The trifucosylated structure provided the most significant difference between NASH-related HCC and cirrhosis samples.

For the ALC-related samples (Fig. 2B), the same trend was observed with markedly lowered non-fucosylated tri- and tetra-antennary *N*-glycans and enhanced fucosylated tri- and tetra-antennary peaks. In addition to the trifucosylated tetra-antennary *N*-glycan, the bifucosylated tetra-antennary peak (*m*/*z* 3316.69) also exhibited a dramatic increase in ALC-related HCC samples, with an abundance of 12.3%, compared to the abundance of 7.2% in ALC-related cirrhosis samples (Table S1). The trifucosylated tetra-antennary *N*-glycan, which was absent in 8 of 19 ALC-related cirrhosis samples and detected only in low abundance (<0.8%) in the rest of the cirrhosis samples, was found to be present in all the ALC-related HCC samples, with an increased abundance of 2.4% (Table S1).

In HCV samples (Fig. 2C), results were similar to those of ALC and NASH groups. The most important difference between HCC and cirrhosis was in the trifucosylated tetra-antennary *N*-glycan, which was 1.3% in HCV-related cirrhosis samples and 3.1% in HCC samples, respectively (Table S1). The second most important difference in glycoform distribution was the ratio of bifucosylated tetra-antennary peak with a mean value of 7.8% in cirrhosis and 13.5% in HCC (Table S1).

### Glycoform distribution of serum AGP

Herein, we employed the ratio, the percentage of a glycan peak area over the total peak area of all glycans, to represent the relative abundance of each glycan in a sample. With this normalization approach, the changes in each *N*-glycan of serum AGP are due to the specific glycosylation alteration rather than protein abundance variation.

In total, we quantified 137 *N*-glycan profiles including 72 HCC cases (17 NASH-, 20 ALC-, and 35 HCV-related), 58 cirrhosis cases (19 NASH-, 19 ALC-, and 20 HCV-related) and 7 healthy controls. The relative abundances of the 7 main *N*-glycans in normal and patients with cirrhosis or HCC of different etiologies are shown in Fig. 2D. Healthy controls expressed the highest level of nonfucosylated bi-, tri- and tetra-antennary *N*-glycans. No trifucosylated tetra-antennary structures were observed in healthy controls. The mean level of bifucosylated tetra-antennary *N*-glycans was only 0.9%. For cirrhosis and HCC cases within the same etiology, a moderate decline in nonfucosylated tri- and tetra-antennary *N*-glycans and a slight increase in mono-fucosylated tetra-antennary structure were observed in HCC compared to the cirrhosis counterparts. Although there was a marked increase in the level of bifucosylated tetra-antennary structures in HCC cases, ANOVA analysis showed that no significant difference existed between cirrhosis and HCC cases. The unique pattern of trifucosylated tetra-antennary *N*-glycan, with a mean value of 0.5%, 0.8%, and 1.3% in NASH-, ALC-, and HCV-related cirrhosis samples, was expressed in the corresponding HCC samples with a higher mean value of 1.1%, 2.4% and 3.1%, respectively, suggesting it may serve as a potential marker to distinguish HCC from cirrhosis cases.

The multifucosylated (two or three fucoses attached) tetra-antennary glycan structures have also been identified in a recent LC-TOF-MS based glycopeptide profiling of serum AGP in patients with HCC, where the glycopeptides containing multifucosylated tetra-antennary *N*-glycans were significantly elevated in HCCs compared to patients with chronic hepatitis^20^.

### Trifucosylated *N*-Glycan of serum AGP

**Figure 3:**
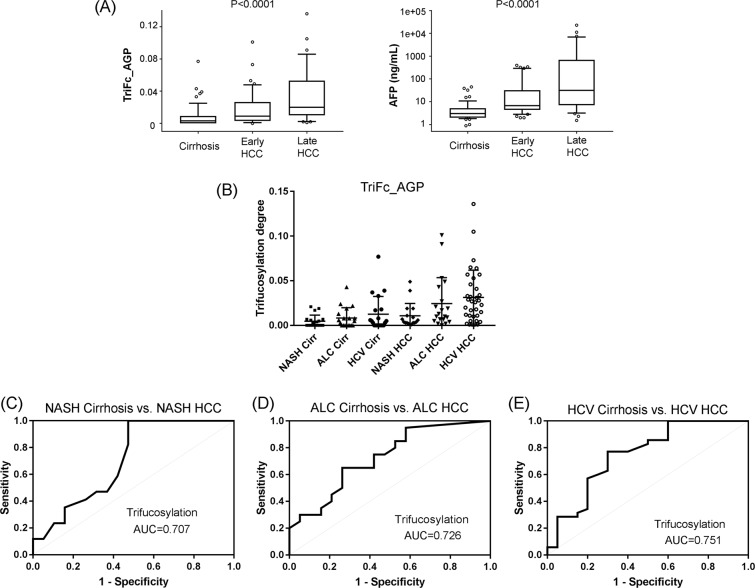
(**A)** Box plots of the trifucosylation degrees of serum AGP and serum AFP levels among patients with cirrhosis, early-stage HCC, and late-stage HCC. The box refers to the 10th and 90th percentile values with a line indicating the median value. Points outside the interquartile range are outliers. The Kruskal-Wallis test showed a significant difference in the trifucosylation degree of AGP and serum AFP level during disease progression from cirrhosis to late HCC (P < 0.0001). **(B)** Scatter plot of the trifucosylation degree of AGP N-glycans in cirrhosis and HCC patients induced by NASH, ALC, and HCV, respectively. **(C–E)** Receiver operating characteristics (ROC) curves of the trifucosylation degree to differentiate HCC from cirrhosis cases induced by NASH (**C**), ALC (**D**), and HCV (**E**) respectively.

We further investigated the distribution of the trifucosylated *N*-glycan in serum AGP among patients with cirrhosis, early HCC, and late HCC, respectively, with a comparison of that of serum AFP levels. Boxplots were used for comparing distributions and identifying outliers (Fig. 3A). The Kruskal-Wallis test showed an overall significant difference for both markers among cirrhosis, early HCCs, and late HCCs (P < 0.0001). In addition, Wilcoxon test showed that the trifucosylated *N*-glycan of serum AGP was significantly increased in late HCC compared to early HCC (P = 0.0033) and cirrhosis (P < 0.0001). In this sample set, serum AFP level also showed a significant increase in early HCC (P = 0.0023) and late HCC (P < 0.0001) when compared to cirrhosis patients.

### Analysis of branching and fucosylation degrees

Changes in glycosylation, such as branching structure and fucosylation, can predict numerous diseases including cancer and immune system deficiencies^33–35^. We therefore investigated whether the alteration in branching degree or fucosylation degree was related to the progression from liver cirrhosis to HCC. The statistical analyses of the branching degree (bi, tri, and tetra) and fucosylation degree (mono-Fc, biFc, triFc and total Fc) of serum AGP are summarized in Table 2 (for all HCC and cirrhosis samples) and Table S1 (for individual etiologies).

**Table 2:** Summary of the fucosylation degree and branching degree of serum AGP as well as clinical factors of AFP and INR in all HCC and cirrhosis samples.

Variable		Diagnosis	N	min	median	max	mean	std dev	P-value
Fucosylation degree*	total Fc	Cirrhosis	58	0.05	0.54	1.18	0.54	0.30	0.0050
		HCC	72	0.15	0.72	1.54	0.72	0.32	
	triFc	Cirrhosis	58	0.00	0.00	0.08	0.01	0.01	<0.0001
		HCC	72	0.00	0.01	0.14	0.02	0.03	
	biFc	Cirrhosis	58	0.00	0.05	0.24	0.07	0.07	0.0003
		HCC	72	0.01	0.10	0.37	0.12	0.09	
	mono-Fc	Cirrhosis	58	0.05	0.42	0.69	0.38	0.17	0.5364
		HCC	72	0.13	0.45	0.57	0.42	0.10	
Branching degree	Bi	Cirrhosis	58	0.03	0.09	0.28	0.11	0.06	0.3549
		HCC	72	0.02	0.09	0.60	0.11	0.09	
	Tri	Cirrhosis	58	0.25	0.45	0.67	0.45	0.08	0.0125
		HCC	72	0.20	0.41	0.66	0.41	0.09	
	Tetra	Cirrhosis	58	0.17	0.45	0.71	0.44	0.11	0.0347
		HCC	72	0.07	0.51	0.77	0.48	0.14	
AFP		Cirrhosis	57	0.00	3.00	45.10	5.74	8.48	<0.0001
		HCC	71	1.50	10.40	22700.00	783.40	3145.59	
INR		Cirrhosis	57	1.00	1.20	1.70	1.19	0.17	0.0266
		HCC	70	0.90	1.10	2.10	1.13	0.19	

*Total Fc, total fucosylation degree of serum AGP; triFc, trifucosylation degree of serum AGP; biFc, bifucosylation degree of serum AGP; mono-Fc, mono-fucosylation degree of serum AGP.

### Branching degrees

The Bi-branching, Tri-branching and Tetra-branching degrees were calculated by the ratio of the sum of abundances of all bi-, tri-, and tetra-antennary *N*-glycans, respectively, to all glycans identified in the sample.

As shown in Table S1, healthy subjects expressed a higher ratio of bi-antennary glycans (17.3%) and a lower ratio of tetra-antennary structures (35.2%) compared to that of cirrhosis and HCC cases. In comparison to cirrhosis cases of the same etiology, there was a small decrease in bi- and tri-branching degree and a small increase in tetra-branching degree in HCC of the ALC and HCV groups, while NASH-related HCC group showed a small increase in bi- and tetra-branching degree and a small decrease in tri-branching degree. The data indicates a slight elevation in branching degree in the progress from liver cirrhosis to HCC; however, it should be noted that in all etiologies, no considerable differences in branching degree were observed between cirrhosis and HCC cases.

In terms of the differences between groups, the NASH group expressed the lowest bi-branching degree (8.6% for cirrhosis and 10.6% for HCC), ALC group expressed the highest ratio of tri-branching degree (48.1% for cirrhosis and 44.3% for HCC) and the HCV group had the highest tetra-branching degree (45.9% for cirrhosis and 48.3% for HCC) similar to that of the NASH group (45.6% for cirrhosis and 47.9% for HCC). There was no apparent distinction in branching degree among NASH, ALC and HCV-related groups.

### Fucosylation degrees

Fucosylation may be an important index to assess the process of malignancy development where fucosylation changes could provide increased proliferative capability and result in subsequent metastasis^36^. The total fucosylation index is defined as^35,37^

$${\rm{Total}}\,{\rm{fucosylation}}\,{\rm{degree}}=(1\times {\rm{glycanF}}1+2\times {\rm{glycanF}}2+3\times {\rm{glycanF}}3)/\sum {\rm{glycans}}$$

where glycanF1 means the sum of abundances of mono-fucosylated glycans including mono-fucosylated bi-, tri- and tetra-antennary *N*-glycans, and glycanF2 means that of bifucosylated glycans. Since the trifucosylated tetra-antennary *N*-glycan is the only trifucosylated structure, glycanF3 refers to its abundance. ∑glycans represents the sum of abundances of all glycans identified in the sample. The scatter plot of the total fucosylation degree of AGP *N*-glycans in healthy subjects and patients with HCC or cirrhosis of different etiologies is shown in the Supplemental Fig. S3.

As shown in Table S1, the mean value of the total fucosylation degree of healthy controls was only 19.6%, whereas this value was markedly increased in cirrhosis patients (42.4~60.6%) and HCCs (52.6~78.4%), suggesting this index can be used to distinguish cirrhosis and HCC cases from healthy subjects. Among NASH, ALC, and HCV groups, the NASH group exhibited the lowest total fucosylation degree of 42.4% for cirrhosis samples and 52.6% for HCC samples, a small increase in HCC cases. For the HCV group, a moderate increase was observed in HCC samples with the mean value of 78.4% compared to 60.6% for cirrhosis samples, which was similar to that of the ALC group (59.5% for cirrhosis and 76.7% for HCC, respectively).

We also calculated other indexes of fucosylation degree including mono-, bi-, and tri-fucosylation degree, which are defined as

$$\begin{array}{rcl}\mathrm{mono} \mbox{-} \mathrm{fucosylation}\,{\rm{degree}} & = & \sum {\rm{glycanF}}1/\sum {\rm{glycans}}\\ \mathrm{bi} \mbox{-} \mathrm{fucosylation}\,{\rm{degree}} & = & \sum {\rm{glycanF}}2/\sum {\rm{glycans}}\\ \mathrm{tri} \mbox{-} \mathrm{fucosylation}\,{\rm{degree}} & = & \sum {\rm{glycanF}}3/\sum {\rm{glycans}}\end{array}$$

Where ∑glycanF1, ∑glycanF2, ∑glycanF3 were defined as mentioned above, and the results of calculation are also included in Table 2 for all HCC and cirrhosis and Table S1 for individual etiologies.

Table 2 displayed the comparison of each marker between HCC and cirrhosis based on the Wilcoxon test. AFP showed significant higher values in HCC patients compared to cirrhosis patients, while INR showed an opposite trend. More importantly, HCC samples yielded statistically significantly higher values in tri-, bi-, and total-fucosylation degrees compared to cirrhosis samples (P-values were <0.0001, 0.0030, and 0.0050, respectively). In the case of branching degree, both Tri- and Tetra-antennary showed the similar trend as fucosylation degrees, and P-values were 0.0125 and 0.0347. We also performed the same comparison in each etiology type. Tri-fucosylation degree values were statistically significantly higher in HCC samples than that in cirrhosis samples in all three etiologies. In HCV patients, bi- and total-fucosylation degrees also showed higher values in HCC than that in cirrhosis samples. Among different etiologies, the NASH group displayed the lowest mono-, bi-, and tri-Fc degree (31.1%, 4.9%, and 0.5% for cirrhosis, and 36.5%, 6.4% and 1.1% for HCC) compared to the ALC and HCV groups (Table S1).

### Diagnostic performance of trifucosylation degree of AGP in each etiology

The scatter plot of the trifucosylation degree of serum AGP in cirrhosis and HCC patients caused by NASH, ALC, and HCV, respectively, is shown in Fig. 3B. Statistical analysis showed a significant difference between HCC and cirrhosis samples in all etiologies (P < 0.0001). Among all etiologies, the trifucosylation degree of AGP had an AUC of 0.750 (95% CI: 0.663, 0.829) in distinguishing HCC from cirrhosis. The receiver operation characteristic (ROC) curves of the trifucosylation degree of AGP between HCC and cirrhosis cases of different etiologies are shown in Fig. 3C–E. The trifucosylation degree of AGP had an AUC of 0.707 (95% CI: 0.532, 0.872) for the NASH group, (Fig. 3C), 0.726 (95% CI: 0.547, 0.872) for the ALC group (Fig. 3D), and 0.751 (95% CI: 0.595, 0.884) for HCV group (Fig. 3E). These data suggest that the trifucosylation degree of AGP may serve as a promising predictor to track the disease progression from cirrhosis to HCC.

The AUC values of the triFc degree of AGP in early HCCs of each etiology are summarized in Table 3. In the particular case of NASH group, the trifucosylation degree of AGP had a better performance in the detection of early NASH-related HCCs than AFP (AUC 0.658 vs. 0.641).

**Table 3:** Summary of ROC analysis for individual markers.

Case	Marker	All		NASH		ALC		HCV	
		AUC	95% CI	AUC	95% CI	AUC	95% CI	AUC	95% CI
*all HCC*	AFP	0.820	(0.749, 0.891)	0.761	(0.585, 0.905)	0.820	(0.658, 0.951)	0.841	(0.729, 0.938)
	INR	0.612	(0.516, 0.702)	0.794	(0.630, 0.918)	0.619	(0.447, 0.781)	0.674	(0.539, 0.811)
	triFc	0.750	(0.663, 0.829)	0.707	(0.532, 0.872)	0.726	(0.547, 0.872)	0.751	(0.595, 0.884)
	biFc	0.684	(0.595, 0.770)	0.633	(0.446, 0.796)	0.642	(0.458, 0.812)	0.714	(0.563, 0.860)
	total Fc	0.644	(0.546, 0.738)	0.610	(0.421, 0.786)	0.608	(0.413, 0.776)	0.665	(0.506, 0.809)
*early HCC*	AFP	0.777	(0.686, 0.863)	0.641	(0.462, 0.831)	0.852	(0.665, 0.990)	0.779	(0.610, 0.906)
	INR	0.598	(0.487, 0.707)	0.785	(0.579, 0.935)	0.667	(0.474, 0.842)	0.665	(0.480, 0.819)
	triFc	0.695	(0.593, 0.785)	0.658	(0.459, 0.842)	0.725	(0.526, 0.895)	0.671	(0.486, 0.838)
	biFc	0.638	(0.528, 0.738)	0.577	(0.371, 0.775)	0.684	(0.481, 0.873)	0.653	(0.450, 0.822)
	total Fc	0.591	(0.470, 0.698)	0.555	(0.354, 0.766)	0.636	(0.421, 0.833)	0.590	(0.400, 0.782)

The AUC values of other variables, including AFP, INR, and (i.e. biFc and total Fc) of AGP, are also listed in Table 3. Differences in total fucosylation were not sufficient to distinguish HCC from cirrhosis of the same etiology, since corresponding AUC values were 0.610, 0.608 and 0.665 for NASH, ALC and HCV groups, respectively (Table 3).

### Combinatorial analysis of trifucosylation degree of AGP with other biomarkers

Among all HCCs, the triFc degree of AGP (triFc_AGP) had an AUC of 0.750 (95% CI: 0.659, 0.828) for differentiating HCC from cirrhosis (Table 4). When combining the triFc_AGP with AFP and INR, the AUC was improved to 0.853 (95% CI 0.781, 0.913) compared to AFP alone (AUC = 0.820; 95% CI 0.747, 0.884). For distinguishing any stage HCC from cirrhosis, AFP alone achieved 72% sensitivity and 79% specificity. Combining AFP with the triFc_AGP increased sensitivity and specificity to 76% and 81%, respectively. The panel of the three markers (triFc_AGP, AFP, and INR) improved sensitivity to 80% while maintaining a specificity of 79%.

**Table 4:** Diagnostic accuracy measurements in all HCCs and early HCCs, respectively, according to ROC curve analysis of the trifucosylation degree of AGP, AFP, INR, and marker panels.

*All HCC vs. Cirrhosis*					
Model	AUC	95% CI	Cutoff*	Sens*	Spec*
1 * AFP	0.820	(0.747, 0.884)	2.722	0.72	0.79
1 * INR	0.612	(0.523, 0.708)	1.138	0.39	0.47
1 * triFc	0.750	(0.659, 0.828)	0.013	0.64	0.76
0.7296 * AFP +12.6855 * triFc	0.832	(0.764, 0.897)	2.121	0.76	0.81
0.9622 * AFP −5.967 * INR	0.837	(0.764, 0.901)	−4.147	0.86	0.71
−5.7676 * INR +39.8998 * triFc	0.780	(0.695, 0.859)	−5.653	0.77	0.77
0.817 * AFP −6.8425 * INR +17.8902 * triFc	0.853	(0.781, 0.913)	−4.960	0.80	0.79
*Early HCC vs. Cirrhosis*					
1 * AFP	0.777	(0.680, 0.865)	2.433	0.77	0.67
1 * INR	0.598	(0.496, 0.709)	1.138	0.40	0.47
1 * triFc	0.695	(0.590, 0.786)	0.013	0.53	0.76
0.6951 * AFP +0.3118 * triFc	0.780	(0.688, 0.870)	1.696	0.77	0.67
0.8585 * AFP −5.9504 * INR	0.793	(0.698, 0.876)	−4.297	0.80	0.71
−3.9602 * INR +26.5163 * triFc	0.699	(0.591, 0.807)	−3.846	0.65	0.79
0.8387 * AFP −6.0249 * INR +2.8025 * triFc	0.795	(0.691, 0.887)	−4.297	0.74	0.77

*The cutoff and its corresponding sensitivity (sens) and specificity (spec) are determined by the point with shortest distance to the point when sensitivity = 1 and specificity = 1. ^+^Marker values were taken log2 transformation when fitting the models.

**Figure 4:**
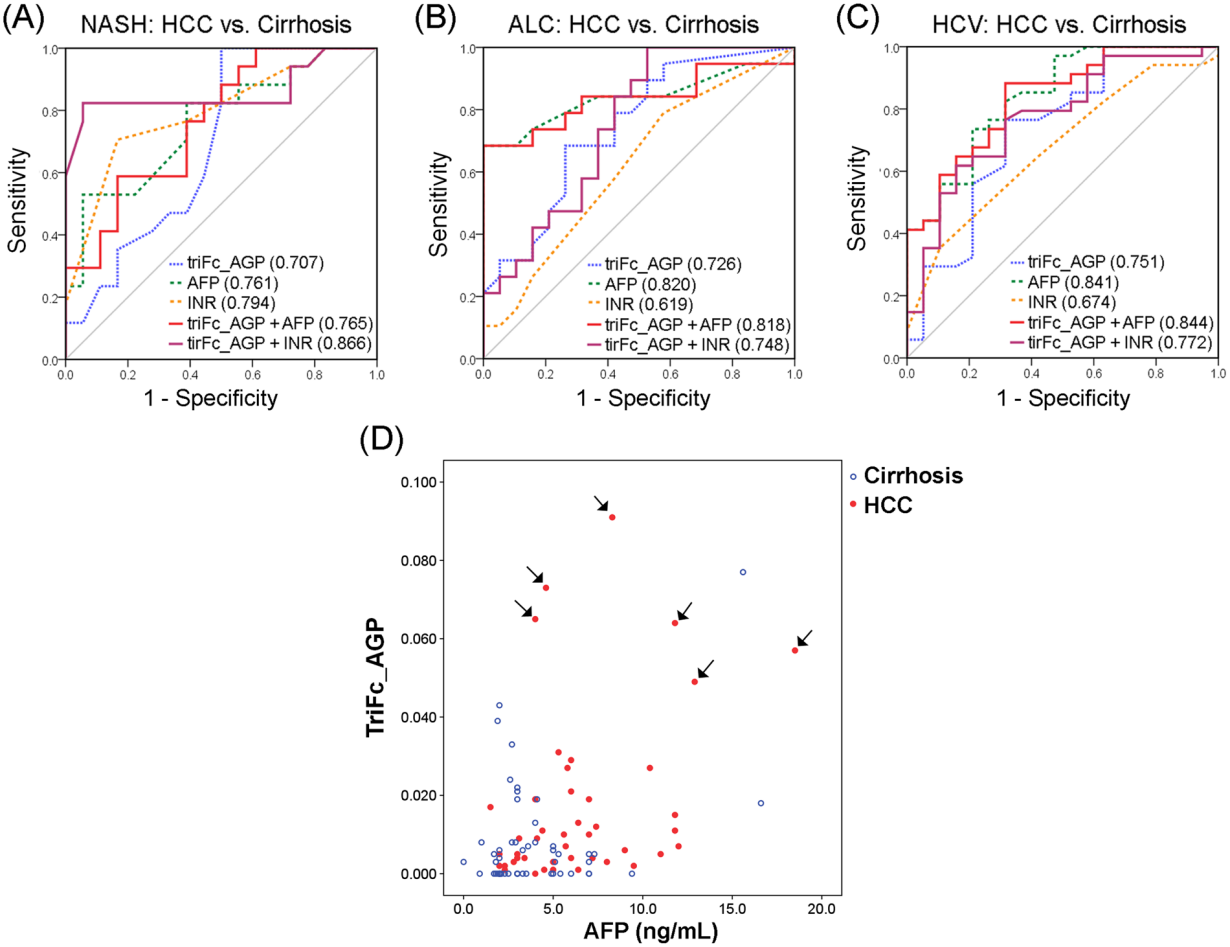
(**A**–**C**) Combined ROC analysis between HCC and cirrhosis induced by NASH (**A**), ALC (**B**), and HCV (**C**), respectively (blue dash line, ROC curve of trifucosylation; green dash line, AFP; orange dash line, INR; red solid line, trifucosylated AGP and AFP combined; purple solid line, trifucosylated AGP and INR combined). **(D)** 2D scatter plot of the trifucosylation degree of serum AGP and the clinical AFP value in cirrhosis (*blue*) and HCC (*red*) patients with AFP < 20 ng/mL. Each spot represents an individual patient. HCC patients with negative AFP but high level of the trifucosylated AGP are marked with arrows.

In each etiology, when combining the trifucosylation degree of AGP with clinical variables, the ROC curves were performed for NASH (Fig. 4A), ALC (Fig. 4B), and HCV (Fig. 4C), respectively.

When combining triFc_AGP, AFP and INR among NASH samples, the combinatorial ROC analysis yields an AUC of 0.882, indicating a significantly improved performance in distinguishing NASH-related HCC from cirrhosis cases relative to AFP alone (AUC 0.761). However, the panel combining triFc_AGP, AFP and INR had a slightly better AUC than AFP alone among the ALC group (0.842 vs. 0.820) and the HCV group (0.880 vs. 0.841).

Notably, among early HCCs in each etiology, the panel significantly improved the AUC with an increase of 28% in early detection of NASH-related HCCs compared to AFP alone (AUC 0.818 vs. 0.641), while it had a slightly better AUC than AFP in the ALC group (0.914 vs. 0.852) and HCV group (0.830 vs. 0.779). The result showed that the panel had the greatest benefit in early detection of the NASH group compared to the ALC and HCV groups. This is a preliminary finding over a limited sample size and a larger cohort of NASH patients will be needed to confirm our findings.

### Performance of trifucosylation degree of AGP in early HCCs

HCC samples included 40 early stage HCC and 32 late stage HCC patients, therefore, we further evaluated the diagnostic accuracy of the trifucosylation degree of AGP for detecting early stage HCC. According to ROC curve analysis, the optimal cutoff value, sensitivity, and specificity of the trifucosylation degree of AGP, AFP, INR, and marker panels are summarized in Table 4. To determine the optimal cutoff value, we chose the point with shortest distance from the ideal cutoff point (sensitivity = 1, specificity = 1).

For differentiating early stage HCC from cirrhosis, the trifucosylation degree of AGP had an AUC of 0.695, with the optimal cut-off resulting in 53% sensitivity and 76% specificity. AFP had an AUC of 0.777, with a sensitivity of 77% and a specificity of 67%. A panel of the three markers (triFc_AGP, AFP, and INR) resulted in an AUC of 0.795 (95%CI: 0.691, 0.887). Based on the optimal cutoff, the panel had an improved specificity of 77% while the sensitivity was 74%, comparable to the performance of AFP alone.

### Performance of trifucosylation degree of AGP in AFP-Negative patients

We also evaluated the diagnostic performance of the trifucosylation degree of AGP in patients with AFP < 20 ng/mL, which are recorded as AFP-negative in the clinic. In this sample set, 61.1% (44 of 72) HCC patients were AFP-negative. Figure 4D shows the 2D scatter plot of the trifucosylation degree of serum AGP and the clinical AFP value in cirrhosis (blue hollow dots) and HCC (red solid dots) patients with AFP negative. As shown in Fig. 4D, the cirrhosis samples were clustered in the lower left panel of the plot, indicating low levels of both the trifucosylation degree of AGP and AFP value. Interestingly, on the upper panel of the plot, 6 AFP-negative HCC patients were found with distinctly increased trifucosylation degree of AGP (marked with arrows). The ROC analysis showed that the trifucosylation degree of AGP had an AUC of 0.709, with 52% sensitivity and 80% specificity. AFP had an AUC of 0.749, with a sensitivity of 77% and a specificity of 65%. When combined with AFP, the trifucosylation degree of AGP yielded an AUC of 0.765, with 61% sensitivity and 85% specificity. The result demonstrated that the trifucosylation degree of AGP can significantly improve the specificity for AFP-negative HCC patients from 65% to 85%, which could serve as a promising marker for monitoring AFP-negative HCC patients.

## Conclusion

Serum AGP is synthesized mainly by hepatocytes in the liver, with approximately 45% of molecular weight contributed by *N*-glycans^14^. Changes in serum AGP glycosylation are highly correlated with HCC progression^19–21^; however, it is unclear how *N*-glycan structures of AGP alter from liver cirrhosis to early HCC in different etiologies. In this study, we performed a comprehensive comparison of *N*-glycan structures of serum AGP between HCC and cirrhosis patients of the three common etiologies (i.e. NASH, ALC, and HCV) in order to determine whether glycosylation patterns of AGP could be used as a marker for early detection of HCC in different etiologies.

The quantitative mass spectrometry-based profiling of *N*-glycans of serum demonstrated a unique trifucosylated tetra-antennary *N*-glycan of AGP which was predominantly identified in HCCs. Notably, this trifucosylated *N*-glycan is a novel structure for AGP, which was not observed in other serum glycoproteins (i.e. IgGs, haptoglobin, A1AT) in glycomic studies. The trifucosylated tetra-antennary *N*-glycan was significantly increased in HCCs compared to patients with cirrhosis, suggesting it could serve as an early detection biomarker for HCC with sufficient biomarker validation. Regardless of the etiologies, the trifucosylated *N*-glycan of AGP (triFc_AGP) could differentiate HCC from cirrhosis regardless of etiology of liver disease and showed improved performance characteristics when combined with other serum markers. Compared to the ALC and HCV groups, the panel of three markers (triFc_AGP, AFP, and INR) had the greatest benefit in detection of NASH-related HCCs compared to AFP alone.

Importantly, triFc_AGP can identify a number of AFP-negative HCC patients, where the ROC analysis showed that the combination of triFc_AGP and AFP can significantly improve the specificity for AFP-negative HCC patients to 85% compared to AFP alone (65%), using the optimal cutoffs. The trifucosylated *N*-glycan of AGP has promising performance characteristics in AFP negative patients. The performance of the potential biomarker panel will require further validation using a large cohort of patients.

## Methods

### Materials

We used commercially available chemicals and reagents for all components of this analysis. Ammonium acetate, chloroform, dimethyl sulfoxide, HPLC-grade acetonitrile (ACN) and water, iodomethane, 2-mercaptoethanol, monoclonal anti-HA agarose, neuraminidase, sodium acetate, trifluoroacetic acid, and 70% perchloric acid were purchased from Sigma-Aldrich (St. Louis, MO). Sodium hydroxide was from Fisher Scientific and sodium dodecyl sulfate (SDS) from AMR-esco. Solid phase extraction disks were from Supelco and graphitized carbon material from Agela Technologies. N-glycosidase F (PNGase F) was purchased from New England Biolabs (Ipswich, MA). The MALDI matrix, 2,5- dihydroxybenzoic acid (2,5-DHB), was purchased from Thermo Scientific (Rockford, IL), Peptide Calibration Standard II from Bruker, and AGP standard protein from Abcam (Cambridge, MA).

### Serum samples

In total, 137 serum samples of HCC, cirrhosis, and healthy subjects were involved in this study, of which 81 were provided by the University Hospital, Ann Arbor, Michigan, and 56 supplied by the UT Southwestern Medical Center, Dallas, Texas. IRB approval for this analysis and transfer of samples between participating institutions was obtained at both the University of Michigan and UT Southwestern Medical Center. The samples were comprised of 7 healthy controls, 72 HCC cases (17 NASH-, 20 ALC-, and 35 HCV-related HCCs), and 58 cirrhosis (19 NASH-, 19 ALC-, and 20 HCV-related cirrhosis). The clinical information of patients with HCC and cirrhosis is shown in Table 1. In addition to AFP, other clinical variables such as ALT, AST, albumin, total bilirubin, INR, and MELD score were available. Samples were aliquoted and stored at −80 °C until further use.

All methods were carried out in accordance with relevant guidelines and regulations. All experimental protocols were approved by the University of Michigan under IRB# 2003-0448 (HUM00046376). Informed consent was obtained from all subjects.

### Workflow

The workflow of this study is summarized in Fig. 1A. A MALDI-MS based glycomic approach was employed to investigate the changes in *N*-glycan patterns of serum AGP between HCC and cirrhosis. First, AGP was purified from patient serum in two steps, i.e. acid precipitation and adsorption by heme agarose (HA) beads. The purified AGP was then deglycosylated and desialylated followed by extraction of *N*-glycans using porous graphitized carbon (PGC) tips. Extracted *N*-glycans were subsequently permethylated to enhance the sensitivity in MS detection and simplify the fragmentation process of *N*-glycans^38^. From the mass data, analyses of branching degree and fucosylation degree were conducted and compared between HCC and cirrhosis patients of different etiologies.

### Measurement of the concentration of serum AGP

The protein level of serum AGP was measured by ELISA assay (Abcam, Cambridge, MA) following the manufacturer’s instructions. Eighty-eight serum samples (36 NASH-, 30 HCV-, 20 ALC-, 2 normal) were involved in the ELISA assay. The absorbance at 450 nm was read on a microplate reader (BioTek, Winooski, VT). The concentration of unknown samples was determined according to the standard curve and then multiplied by the value of the dilution factor.

### Purification of AGP using acid precipitation

We purified AGP from patient sera using a pH based precipitation method^32^ with some modification. This method enabled efficient isolation of AGP from high abundance blood proteins without the use of an antibody for affinity capture.

Serum samples were placed on ice and thawed immediately before use. 1.2 M perchloric acid was added to 20 μL of serum at a 1:1 (v/v) ratio, which can precipitate most of the impurity proteins while AGP remains in the liquid phase. The resulting 0.6 M perchloric acid concentration provided optimum recovery of AGP with minimal contamination by other proteins^32^. Partial desialylation may occur at this concentration of perchloric acid, but Schultze *et al*. demonstrated that no sialic acid is released from AGP after short exposure to HClO_4_ at low temperatures^39^. We performed this procedure at low temperature (4 °C) and decreased the exposure time to HClO_4_ to prevent this cleavage.

The mixture was vortexed for 20 s and then centrifuged at 3000 × g for 20 min at 4 °C to remove the precipitated proteins. The supernatant was pipetted, and transferred to another tube followed by the addition of 0.5 M NaOH solution to neutralize the acidic media. Desalting was achieved by using a 0.5 mL of Zeba desalting spin columns, 7 K MWCO (Thermo Scientific, Rockford, IL) and then the sample was dried in a SpeedVac concentrator (Thermo).

### Depletion of hemopexin

The purity of crude AGP was evaluated by gel electrophoresis where most of the samples exhibited only a single band corresponding to AGP; however, for some samples, a minor contaminant protein (~66 kDa) was observed and further identified as hemopexin by LC-MS/MS analysis. As a heme-binding protein, hemopexin can be absorbed by heme agarose (HA) beads. Therefore HA beads were used to remove hemopexin. 25 μL of HA beads (Sigma) were added into a Pierce Spin Column and centrifuged at 1000 × *g* for 1 minute, followed by two washes with 200 μL of Coupling Buffer. Then the crude AGP, dissolved in 55 μL of Coupling Buffer, was added to the column and incubated at 4 °C for 24 h to ensure the thorough adsorption of hemopexin to the HA beads. After centrifugation, the flow-through fraction was the purified AGP whose purity was confirmed by gel electrophoresis. The purified AGP was desalted and dried down.

### Deglycosylation and desialylation of AGP

*N*-glycans were released from serum AGP using PNGase F as reported previously^37^. Briefly, purified AGP was dissolved in 9 μL water with 1 μL denaturing solution (0.2% SDS, 100 mM 2-mercaptoethanol) and incubated at 60 °C for 30 min. Ammonium bicarbonate was added to reach a final concentration of 15 mM. Two units of PNGase F were added to the sample and incubated at 37 °C for 14 h followed by heating at 95 °C for 10 min to quench the reaction. The sample was then dried down in the SpeedVac concentrator.

Because the presence of sialic acids increases the heterogeneity of *N*-glycans and hence complicates the corresponding spectrum, the sialic acids were removed from *N*-glycans using neuraminidase. Briefly, the sample was redissolved in 30 μL of 20 mM ammonium acetate with 40 mU of neuraminidase. After incubation at 37 °C for 16 h, the reaction was quenched by heating at 95 °C for 10 min. In-house packed porous graphitized carbon (PGC) tips were employed to extract glycans from the mixture. The extracted glycans were then dried thoroughly.

### Permethylation of *N*-glycans

After extraction with PGC tips, the desialylated glycans were subjected to permethylation which further increases the sensitivity of *N*-glycans to MS analysis. As described previously^37^, the extracted glycans were dissolved in 20 μL of DMSO, into which were sequentially added 3 mg of NaOH powder, 3.8 μL of methyl iodide and 0.2 μL of water. After mixing at room temperature for 10 min, 24 μL of ice-cold water was added to the mixture, and then an equal volume of chloroform was added to extract the permethylated glycans. The organic layer was washed repeatedly with water until neutral pH. Finally, the organic phase was dried under vacuum to provide permethylated glycans.

### MALDI-TOF analysis

MALDI-MS analysis of permethylated glycans was performed on an AutoFlex Speed MALDI instrument (Bruker), while further confirmation of glycan composition was achieved by CID MS/MS fragmentation on an Axima MALDI-QIT-TOF instrument (Shimadzu). Briefly, permethylated glycans were reconstituted in 2 μL of 20% acetonitrile. 0.5 μL of samples were spotted on the MALDI plate and allowed to air dry. 0.5 μL of DHB matrix (10 mg/mL DHB in 50% acetonitrile with 1 mM sodium acetate) was spotted and allowed to air dry. The mass accuracy with calibration was 30 ppm. 1 nmol/μL peptide mixtures including angiotensin II (*m/z* 1046.54), angiotensin I (*m/z* 1296.68), substance P (*m/z* 1347.74), bombesin (*m/z* 1619.82), ACTH 1–17 (*m/z* 2093.09), ACTH 18-39 (*m/z* 2465.20), and somatostatin 28 (*m/z* 3147.47) were used for instrument calibration. All glycans were sodiated and analyzed in the positive ion mode in this study. Glycan composition was predicted with Glycoworkbench and further confirmed by collision-induced dissociation (CID) MS/MS analysis. Only glycan structures included in the GlycoSuite database were selected.

### Data analysis

The MALDI MS data were acquired and processed in FlexAnalysis software (Bruker) and *m*/*z* values, intensities, peak area and other data were exported as Excel files. The peak area of each glycan was the addition of both fully permethylated and underpermethylated glycan peaks (14 Da lower than the fully), which was then normalized to the sum of all glycan peak areas and represented as a percentage of the total peak area. Analysis of branching degree and fucosylation degree was conducted by Excel and visualized with Prism 7 (La Jolla, CA). Summary statistics are used to describe the patient characteristics. Discrete variables such as gender are summarized using frequency tables and continuous variables are summarized using median, mean and standard deviation. Boxplots are used to illustrate the marker distributions. Wilcoxon test or Kruskal-Wallis test are used to compare the value of continuous variables across groups.

Logistic regression model is used to combine trifucosylation of AGP with AFP and other labs in the marker panel development. Receiver operation characteristic (ROC) curves are constructed. The area under the curve (AUC) is calculated, and its 95% confidence interval (CI) is estimated using the bootstrapping method. All the analyses are performed using R statistical software (https://cran.r-project.org).

## Supplementary information

Supplemental Information
