# Supplementary material for: Evaluation of AGP Fucosylation as a Marker for Hepatocellular Carcinoma of Three Different Etiologies
Source: Sci Rep. 2019 Aug 9;9:11580. doi: 10.1038/s41598-019-48043-1 (PMC6689003; doi:10.1038/s41598-019-48043-1)
Supplement: Supplementary file 1 — Supplemental Information [file 41598_2019_48043_MOESM1_ESM.pdf]

# Evaluation of AGP Fucosylation as a Marker for Hepatocellular Carcinoma of Three Different Etiologies

*Jing Liang[1,2,§], Jianhui Zhu[1,§], Mengmeng Wang[1,3], Amit G. Singal[4], Mobolaji Odewole[4], Sofia Kagan[4], Veronica Renteria[4], Suyu Liu[5], Neehar D. Parikh[6], and David M. Lubman[1]**

[1]Department of Surgery, University of Michigan, Ann Arbor, MI 48109, USA

[2]School of Chemical Engineering and Technology, China University of Mining and Technology, Xuzhou, Jiangsu 221116, China

[3]Department of Emergency Medicine, Jinling Hospital, Medical School of Nanjing University, Nanjing, Jiangsu 210002, China

[4]Department of Internal Medicine, University of Texas Southwestern Medical Center, Dallas, TX 75390, USA

[5]Department of Biostatistics, University of Texas MD Anderson Cancer Center, Houston, TX 77030, USA

[6]Department of Internal Medicine, University of Michigan, Ann Arbor, MI 48109, USA

* To whom correspondence should be addressed. David M. Lubman, Department of Surgery, University of Michigan Medical Center, 1150 West Medical Center Drive, Building MSRB1, Rm A510B, Ann Arbor, MI 48109-0656. Tel: 734-647-8834. Fax: 734-615-2088. Email: dmlubman@umich.edu

**Supplemental Information**

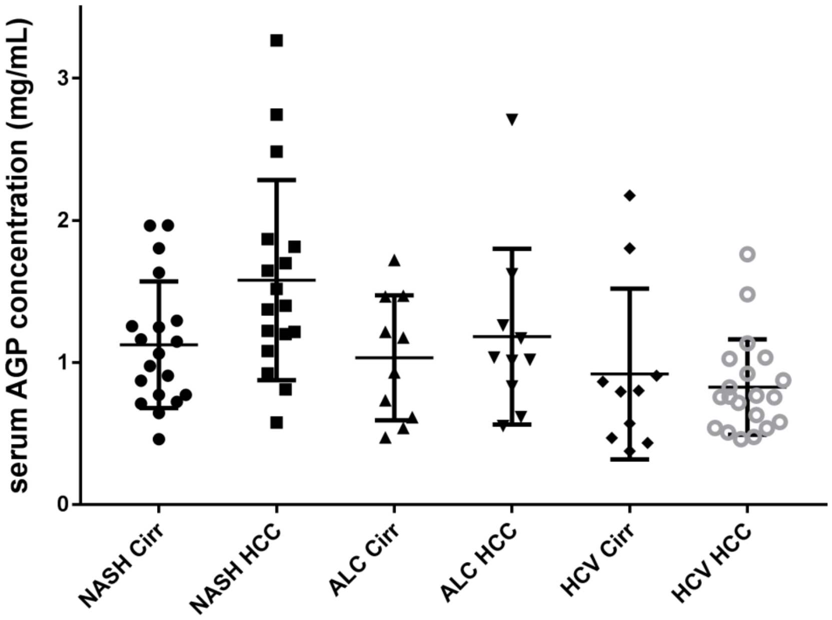


**Figure S1.** Scatter plot of serum AGP concentration measured by ELISA assay in patients with HCC or cirrhosis divided by the etiology, NASH, ALC, and HCV, respectively.

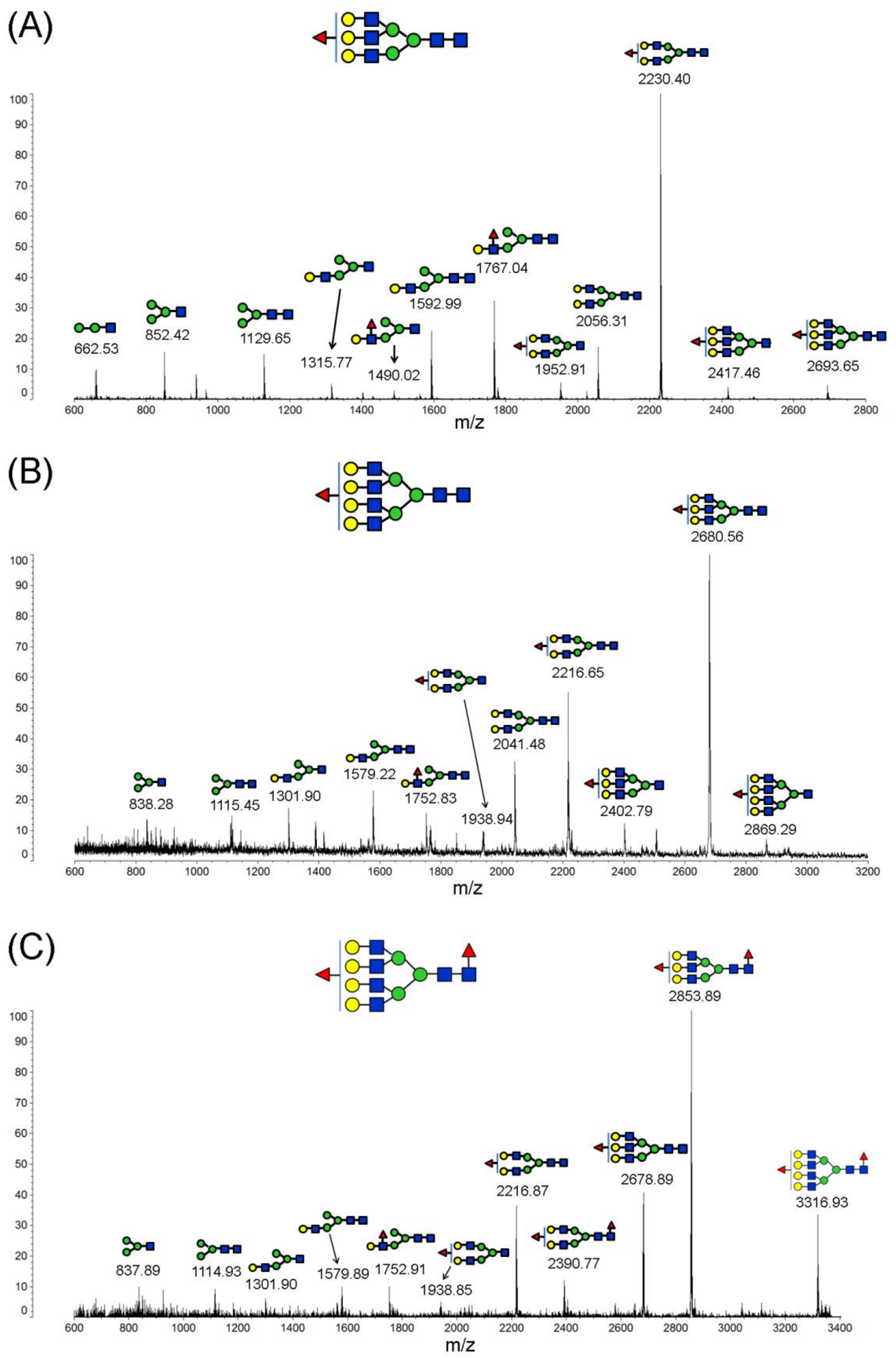


**Figure S2.** Representative MALDI-QIT-TOF MS/MS spectra of glycans at (A) *m/z* 2693.65, (B) *m/z* 3142.68, and (C) *m/z* 3316.93 for glycan composition and core/antennary fucosylation assignment. Fragment ions are labeled with potential structures. (A) The mono-fucosylated tri-antennary glycan (*m/z*

2693.65) is confirmed as antennary fucosylated, where a diagnostic peak at *m/z* 1490.02 is the cleavage product of the peak at *m/z* 1767.04 after loss of core GlcNAc, indicating no core fucose attached originally. (B) Fragment ions at *m/z* 1938.94, 2402.79, and 2869.29 indicate antennary fucosylation for the mono-fucosylated tetra-antennary glycan (*m/z* 3142.68). (C) The bifucosylated tetra-antennary glycan (*m/z* 3316.93) is determined with both core and antennary fucosylation by the presence of a fragment ion at *m/z* 1938.85 which is the product of the ion at *m/z* 2390.77 after loss of core Fuc-GlcNAc, with one fucose attached to the antennae.

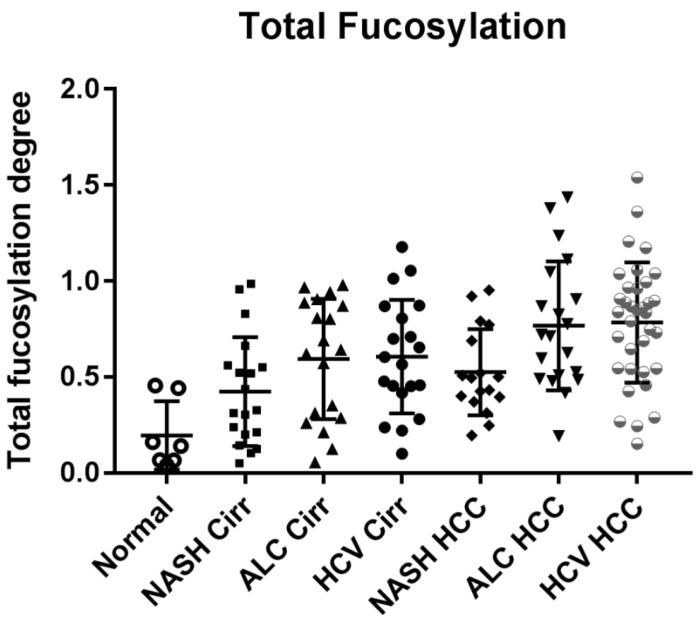


**Figure S3.** Scatter plot of the total fucosylation degree of AGP *N*-glycans in healthy subjects and patients with cirrhosis or HCC caused by NASH, ALC, and HCV, respectively.

**Table S1.** Branching degree and fucosylation degree of serum AGP in healthy control, HCC and cirrhosis patients of different etiologies.

| | | Branching Degree (%, mean) | | | Fucosylation Degree (%, mean) | | | |
|---|---|---|---|---|---|---|---|---|
| | | **Bi** | **Tri** | **Tetra** | **mono-Fc** | **bi-Fc** | **tri-Fc** | **total Fc** |
| **Healthy Control** | | 17.3 | 47.6 | 35.2 | 17.6 | 0.9 | 0.0 | 19.6 |
| **Cirrhosis** | NASH | 8.6 | 45.8 | 45.6 | 31.1 | 4.9 | 0.5 | 42.4 |
| | ALC | 10.8 | 48.1 | 41.1 | 42.6 | 7.2 | 0.8 | 59.5 |
| | HCV | 12.2 | 41.9 | 45.9 | 41.3 | 7.8 | 1.3 | 60.6 |
| **HCC** | NASH | 10.6 | 41.5 | 47.9 | 36.5 | 6.4 | 1.1 | 52.6 |
| | ALC | 9.4 | 44.3 | 46.2 | 44.7 | 12.3 | 2.4 | 76.7 |
| | HCV | 11.7 | 39.9 | 48.3 | 42.1 | 13.5 | 3.1 | 78.4 |
